# Immunity, atherosclerosis and cardiovascular disease

**DOI:** 10.1186/1741-7015-11-117

**Published:** 2013-05-01

**Authors:** Johan Frostegård

**Affiliations:** 1 Institute of Environmental Medicine, Unit of Immunology and Chronic Disease, Nobels väg 13, Stockholm, 171 77, Sweden

**Keywords:** Immunity, Atherosclerosis, Cardiovascular disease, Phosholipids, Natural antibodies, T-cells, B-cells, Inflammation

## Abstract

Atherosclerosis, the major cause of cardiovascular disease (CVD), is a chronic inflammatory condition with immune competent cells in lesions producing mainly pro-inflammatory cytokines. Dead cells and oxidized forms of low density lipoproteins (oxLDL) are abundant. The major direct cause of CVD appears to be rupture of atherosclerotic plaques. oxLDL has proinflammatory and immune-stimulatory properties, causes cell death at higher concentrations and contains inflammatory phospholipids with phosphorylcholine (PC) as an interesting epitope. Antibodies against PC (anti-PC) may be atheroprotective, one mechanism being anti-inflammatory. Bacteria and virus have been discussed, but it has been difficult to find direct evidence, and antibiotic trials have not been successful. Heat shock proteins could be one major target for atherogenic immune reactions. More direct causes of plaque rupture include pro-inflammatory cytokines, chemokines, and lipid mediators. To prove that inflammation is a cause of atherosclerosis and CVD, clinical studies with anti-inflammatory and/or immune-modulatory treatment are needed. The potential causes of immune reactions and inflammation in atherosclerosis and how inflammation can be targeted therapeutically to provide novel treatments for CVD are reviewed.

## Background

Atherosclerosis is the dominant cause of cardiovascular disease (CVD) including myocardial infarction (MI), heart failure, stroke and claudication. Atherosclerosis is mainly located in the intima of many middle sized and large arteries, especially where the vessels divide. Most likely this is influenced by the nature of the blood flow, since areas exposed to normal shear stress seem to be protected; here endothelial cells express atheroprotective genes [1]. Also, the adventitia may play a role in atherosclerosis development, and is characterized by lymphocyte infiltrates [2].

Activated endothelium with expression of adhesion molecules appears to be an early event in atherosclerosis, allowing mononuclear leukocytes, such as monocytes and T-cells, to attach to the endothelium and penetrate into the intima. Though not as common as these cells, dendritic cells, mast cells and a few neutrophils and B-cells may also be present in lesions. Another cell type which is present in lesions is smooth muscle cells (SMC) which change phenotype into synthetic SMC and migrate into the intima from the media. The notion of atherosclerosis as an inflammatory disease is based on the finding that immune competent cells are abundant in atherosclerotic lesions, and also are producing cytokines, especially proinflammatory cytokines [3].

The role of immunity, as defined by the role of activated T-cells and B-cells, in atherosclerosis is much less known, especially in humans, although novel data indicate that underlying immunological factors predispose to inflammation in humans and that immune modulation altering atherosclerosis is possible in animal models, especially mice [4]. It is, therefore, important to discuss inflammation in atherosclerosis in the same context as immunity.

Even though atherosclerosis *per se* could decrease blood flow through stenosis and thus induce CVD, the major mechanism appears to be atherothrombosis, usually when plaques are damaged through the effects of proinflammatory cytokines and chemokines on the fibrous cap. When plaques are damaged and rupture, prothrombotic material is exposed to the coagulation system, with ensuing inhibition of blood flow and thus induction of CVD. The major risk factors which can be modified for atherosclerosis (and CVD) are hypertension, smoking, diabetes and dyslipidemia. In addition, age and male sex are of major importance [4].

### Potential causes of inflammation and immune reactions in atherogenesis and plaque complications

Even though both genetic and epigenetic factors influence atherogenesis and risk of CVD, this review is focused on what is perceived as the major potential direct causes of the inflammation and immune reactions in this context.

#### Oxidized LDL and related compounds

Low density lipoprotein modified by oxidation or enzymatic modification (OxLDL) is present at an early stage. Low density lipoprotein (LDL) penetrates into the intima at the earliest stages of atherosclerosis and binds to the proteoglycan matrix, enabling further modification through oxidation and/or enzymatic modification (OxLDL). Also at later stages, oxLDL and related compounds are ubiquitous in lesions [5,6]. Therefore, oxLDL could play a role both in atherogenesis and in plaque complications. OxLDL is immunogenic and activates endothelial cells, monocytes/macrophages and T cells [7-9]. Further, oxLDL is toxic at higher concentrations and could thus be a cause of cell death in lesions [7-9]. Enzymatically modified LDL could play a major role, and phospholipase 2 (PLA2), which causes such modification, is expressed in both normal arteries and atherosclerotic lesions [10] and can induce activation of dendritic cells [11]. The proinflammatory and immune stimulatory effects of oxLDL are mimicked by inflammatory phospholipids, such as lysophosphatidylcholine (LPC), which is a major phospholipid in atherosclerotic lesions [12,13]. Other proinflammatory phospholipids implicated in oxLDL, such as LPC, have phosphorylcholine (PC) as an important epitope, which cause these different phospholipids, to different degrees, to interact with the platelet activating factor (PAF)-receptor, which is one mechanism by which oxLDL exerts its effects [14,15]. Other mechanisms include interaction with Toll-like receptors and scavenger receptors [16,17].

Not only oxidized and/or enzymatically modified phospholipids are implicated as causes of oxLDL’s pro-atherogenic and pro-inflammatory effects; there are several other possibilities. It has even been suggested that epitopes, such as those exposed during LDL-modification and/or oxidation, represent an evolutionary conserved system of danger-associated molecular patterns (DAMP) in parallell with pathogen-associated molecular patterns (PAMP) [17]. One important example of DAMP in addition to phospholipid (PL)-related epitopes, such as PC, is malondialdehyde (MDA) which is generated during oxidation of LDL. MDA forms adducts on proteins and peptides, carbohydrates and DNA [17].

Modified and oxidized apolipoprotein B (apoB) and cholesterol could also be implicated [17] although putative mechanisms are not as well described as for PC-exposing epitopes.

While much evidence from epidemiological studies indicates that smoking is associated with atherosclerosis and CVD [18,19], the exact mechanisms by which smoking could cause inflammation in the arteries are not fully elucidated, although increased oxidation of lipids is one interesting possibility [20]. Interestingly, smoking is associated with increased lipid oxidation [20]. Different animal models of smoking and atherosclerosis indicate that smoking promotes atherogenesis [21-23], and one underlying mechanism is reported to be oxidative stress [24].

In support of inflammatory phospholipids as causes of atherosclerosis are data from clinical studies, where we reported that in conditions with increased atheroslerosis, such as hypertension and systemic lupus erythmatosus (SLE), PC-exposing LDL is increased [25].

Oxidation of LDL, and also increased oxidative stress in diabetes, could promote atherogenesis [26]. Formation of advanced glycation end products (AGEs) could contribute to atherosclerosis (and CVD) in diabetes since AGEs have proinflammatory and potentially atherogenic properties [27-29].

#### Dead cells

The role of cell death as a cause of inflammation in atherosclerosis and plaque rupture is complicated and depends most likely on stage of disease, and naturally, on whether cell death is organized as in apoptosis, or not, as in necrosis. It is likely that a defective apoptotic clearance and ensuing necrosis could contribute to inflammation.

Dying cells can activate the innate immune system and induce an inflammatory response with release of the proinflammatory cytokine IL-1beta, activating the inflammasome [30].

According to the danger hypothesis, endogenous factors (DAMPs) released during cell death induce inflammation. DAMPs include high-mobility group protein B1 (HMGB-1), double-stranded DNA, amyloid-β-peptide and heat-shock proteins (HSP) [31]. Even though cell death is not an early event in atherogenesis (fatty streak formation and infiltration of monocytes/macrphages and T cells apparently come first), it could play a role at a later stage to promote inflammation. Whether it plays a role in plaque rupture is possible but not proved.

#### Phospholipid-related epitopes

Antibodies against phospholipids (aPL), especially against cardiolipin (aCL) are well known to be associated with CVD, especially in SLE patients but it has been difficult to find unequivocal evidence of aPL as atherogenic, and there are both negative and positive reports [25]. In a recent study, we did not find an association between aPL and prevalence of atheroslerotic plaques in patients with SLE [32]. Typical of pathogenic aPL is that they are dependent on plasma co-factors, such as beta2-glycoprotein I (beta2GPI) , to promote CVD. Mechanisms could include a direct effect on endothelium and also interference with anti-coagulant proteins such as Annexin A5 [32,33].

CL has a unique double structure, with four fatty acid chains and is present in bacteria and the inner mitochondrial membrane of eukaryotic cells [34], which is interesting since mitochondria apparently have a bacterial origin [35]. In contrast to aCL, we recently reported that antibodies against oxidized forms of CL (aOxCL) are negatively associated with CVD, low levels giving high risk and high levels low risk [36]. In contrast to aCL, aOxCL are not beta2GPI-dependent [36]. One mechanism could be inhibition of binding and uptake of oxLDL in macrophages [36]. Further, aOxCL and also antibodies against oxidized phosphatidylserine (aOxPS) are negatively associated with atherosclerosis development (unpublished data).

Another example of natural antibodies is those against PC (anti-PC). Several lines of evidence imply that anti-PC could play a role in atherogenesis, both from animal studies, other experimental studies and from clinical cohort studies [37]. Immunization in a mouse model with pneumococcae caused a decrease in atherosclerosis development in parallell with an increase in anti-PC levels, among other antibodies [38]. Both passive and active immunization with PC ameliorates atherosclerosis in mouse models [39,40]. We have reported in several papers that immunoglobulin M (IgM) anti-PC is negatively associated with atherosclerosis development and risk of CVD. Typically, low levels give rise to increased risk and, in some cases, high levels were also associated with decreased risk [37]. We reported for the first time that anti-PC is a protective marker for atherosclerosis development in humans which was also the case with antibodies against malone dialdehyde LDL (anti-MDALDL) and anti-OxLDL [41].

Mechanisms by which human anti-PC could ameliorate atherosclerosis and CVD include: anti-inflammatory effects, inhibition of pro-inflammatory effects by inflammatory phospholipids [42], inhibition of uptake of oxLDL through scavenger receptors [43] and inhibition of cell death induced by LPC, a major inflammatory phospholipid [44]. In another paper, the inflammatory effect of anti-PC is also confirmed in a mouse model, in which facilitating phagocytosis is described as one mechanism. It is possible that natural IgM, such as anti-PC, could counter atherosclerosis development by binding to dead and dying cells in the lesions, increasing phagocytosis and clearance of obnoxious pro-inflammatory compounds [45].

Low levels of anti-PC could thus be a cause of the inflammation in atherosclerosis, although it is less clear by what mechanisms. We have suggested that a Western life style could contribute, and one underlying factor could be some types of infections which are not prevalent in the West which could raise anti-PC [46]; another could be factors which are relatively new from an evolutionary point of view such as gluten [47]. Genetic factors could also play a role in addition to environmental influences since heritability of anti-PC is 37% [48].

#### Heat shock proteins

HSPs, especially HSP60 but potentially also other ones, such as HSP70 and HSP90, represent another interesting potential cause of inflammation in atherosclerosis. This could be of great interest since HSPs are immunogenic and T-cell clones recognizing HSP60 are present in both early and late atherosclerotic plaques [49,50]. HSPs may also activate immune reactions through cross-reactivity with HSP from microorganisms, such as bacteria. This notion is supported by both clinical data with associations between antibodies against HSP60/65 and atherosclerosis, and experimental data where immunization with HSP60/65 aggravates atherosclerosis [51,52].

HSPs could promote inflammation by other mechanisms as well. Besides being specific T-cell antigens *per se*, present on antigen presenting cells, HSP and/or peptides thereof could promote immune activation by other mechanisms. HSPs are chaperones and can form immune complexes with other antigens including tumor-derived ones, and these can be presented through class I or class II antigen presenting pathways [53]. HSP can be passively released which may occur in cell necrosis but also actively through exosomes. HSPs could thus play a role in the extracellular space where they could be endogenous ligands, activating the innate immune system, through Toll-like receptors or by association with endotoxin [54]. The mechanisms by which hypertension could cause inflammation in the artery wall are not clear. One possibility is a direct effect on the endothelium, which could become dysfunctional as a response to injury leading to proinflammatory changes [55]. We suggested in previous studies that hypertension could cause inflammation by induction of immunogenic HSP60/65, which is also induced by oxLDL [56,57]. We reported that HSP70 is a protective factor for development of atherosclerosis among hypertensives, but a putative underlying mechanism is not clear [58].

#### Infections

Infections have been much discussed as potential causes of immune activation and inflammation in atherosclerosis. In early studies, before the rise of the lipid hypothesis, pathologists and others who observed the lesions thought they could be of infectious origin based on the microscopic and macroscopic features of atherosclerosis.

Among the most promising candidates that are present in plaques, promote atherosclerosis in animal studies and have associations with disease in humans are *Chlamydia pneumoniae* (CP), periodontal organisms including *Porphyromonas gingivalis* (PG) and *Aggregatibacter actinomycetemcomitans* (AA), *Helicobacter pylori* (HP) and cytomegalovirus (CMV) [59].

One important starting point underlying the hypotehsis that infections play a role in atherosclerosis was early studies of CP, demonstrating the presence of this pathogen in atherosclerotic plaques [60] and an association between cardiovascular and antibody titer [61-63] by P Saikku and coworkers and others. As is the case with other pathogens, also discussed, such as CMV, there are also studies in which no such associations were demonstrated [59].

A more formal test of the hypothesis has been done with treatment with antibiotics which have an effect on CP. However, four large studies were not positive and did not support the notion of a causative effect of CP on CVD [64-66]. However, there could be other reasons for the negative results. One is that chronic CP may be difficult to treat irrespective of CVD; another is that the treatment trials have been performed on patients with late stage disease, and it is possible that earlier stages would be more responsive [59].

Periodontal microorganisms, such as PG and AA, are also interesting candidates. Even though clinical/epidemiological studies show associations, there are many confounding factors which are difficult to control for, maybe more for these agents than is the case with other pathogens in this context. A recent scientific statement from the American Heart Association supports an independent association between periodontal disease but available data do not support causation, although intervention does decrease systemic inflammation and improves endothelial function [67].

CMV belongs to the Herpes virus group which is very common in the general population; this makes studies of associations difficult to interpret. There are interesting reports of an association between active CMV infection and transplant complications including vasculopathy [68]. A causal relationship between CMV infection and transplant vascular complications seems to be more plausible than associations with atherosclerosis *per se*. CMV is reported to be present in atherosclerotic lesions in many but not all studies [59]. Of note, CMV has also been documented in healthy arteries [69] which could also be taken as an indication that CMV may be an innocent by-stander. On the other hand, there are interesting properties in CMV which could make it a plausible candidate to be a contributing factor in atherosclerosis. For example, CMV infection induces migration of arterial smooth muscle cells [70].

HP, a well known cause of gastritis and gastric ulcer, may also be implicated in CVD and atherosclerosis, although animal experiments are less clear than is the case with CP. Viable bacteria have not been unequivocally demonstrated from lesions. However, there are positive reports of a reduction of CVD after eradication of HP. HP does not promote a local inflammatory reaction to the same extent as do CP, CMV and several other implicated pathogens [59]. Of note, viable HP have not been isolated from atherosclerotic plaques, and mouse experiments have not given any clear indication that HP is a cause of atherosclerosis [71].

Interestingly, treatment regimens for both CP and HP had a positive effect on clinical CVD events [72]. However, as discussed, larger studies for CP were not successful and are needed also for HP.

Other infectious agents that have been discussed and reported as potential causes of CVD and atherosclerosis include HIV, Epstein-Barr virus (EBV), influenza, *Mycoplasma pneumoniae* and *Streptococcus pneumoniae*. Another interesting case, in which there is evidence from human studies, is Borrelia [73], although experimental data or plaque data are not available to the best of my knowledge. However these appear to be less supported by evidence; especially, there is no convincing data from human studies [59].

Taken together, even though the infectious hypothesis in CVD and atherosclerosis has been studied for some decades, there is still little direct, though rather much circumstantial, evidence of a causative role of infectious agents.

Another interesting development, in which infections and atherosclerosis/CVD could be related in a more indirect way, is recent findings implicating the intestinal bacterial flora [74,75]. Intestinal microbiota metabolism of choline and phosphatidylcholine produces trimethylamine (TMA), which is metabolized to proatherogenic trimethylamine-N-oxide (TMAO). Recently, it was demonstrated that metabolism by intestinal microbiota of dietary l-carnitine in red meat produces TMAO and accelerates atherosclerosis in mice. This finding suggests another interesting link between gut flora and atherosclerosis [76].

Another aspect of immunity and inflammation that could be relevant for atherosclerosis is modulation of the host by microorganisms in order to promote their own survival. PC has a central role, being exposed on different types of pathogens including Gram-positive and –negative bacteria, nematodes and protozoa. In this context, the pathogens use PC to create a favorable environment for themselves in the host. One interesting possibility is therefore that PC exposed in the atherosclerotic plaque in fact modulates the local immune reaction into an unresolved chronic inflammation, in a similar manner as PC-exposing pathogens do in chronic infections [77].

Further research is needed to clarify if the described associations are causative in humans.

#### Other types of inflammation having a potentially causative role in atherogenesis

Even though it remains to be demonstrated that infections play a direct role in atherogenesis, they could still be of great importance indirectly. By being present in lesions, they could stimulate an ongoing inflammatory process, which in the long run may lead to increased atherosclerosis and ensuing CVD. Further, it is possible that the total infectious burden could be a risk factor for increased atherosclerosis and CVD, possibly through promotion of systemic inflammation, platelet aggregation and endothelial dysfunction, which in turn could influence atherogenesis [59,78].

Raised levels of C-reactive protein (CRP) have been implicated as a risk marker for atherosclerosis and CVD in many studies, although it is not clear if CRP could play a causative role. Also, cytokines, such as IL-6, raised systemic levels of oxLDL and have been implicated in many studies [4]. Another example of an interesting group of inflammatory compounds is lipid mediators, such as leukotrienes, which are present in advanced atherosclerotic plaques [79,80].

Another interesting example of indirect effects by other types of inflammation is the relationship between chronic inflammatory and autoimmune diseases and atherosclerosis (and CVD). Here the evidence is strongest for the prototypic autoimmune disease SLE where the risk of CVD is very high. According to one report the risk increased 50 times [81] and several other studies have also reported a high risk (although from low levels since young and middle aged women without SLE (or familial hyperlipidemia) very seldom develop CVD, and, even less, advanced atherosclerosis [25]. Also in rheumatoid arthritis (RA), an increased prevalence of atherosclerosis has been reported, although the evidence appears less clear than in SLE. In other rheumatic diseases, an increasing number of papers indicate an increased atherogenesis [25]; which is confirmed in a recent meta-analysis which demonstrates that rheumatic diseases increase the risk of atherosclerosis [82].

It is interesting to note that this discussion is not new. Already in the first half of the 19th century, the legendary pathologists Rokitansky and Virchow (the former as the first) reported that atherosclerosis is an inflammatory process although their opinions differed somewhat, since Rokitansky thought inflammation in atherosclerosis was a secondary phenomenon, but Virchow proposed it was a primary factor [83]. Non-traditional risk markers, such as low anti-PC and inflammation *per se* appear to play a role, in addition to traditional ones, such as hypertension, dyslipidemia and to a varying degree, smoking [25].

Although opinions may differ somewhat as to what comes first in the development of atherosclerosis, it appears likely that different factors could act in concert. It is also possible that there are differences between different individuals, where some may have atherosclerosis with more inflammation than others. It is also possible that infection could play an important role among subgroups of individuals and patients.

Recently, new aspects of immune mechanisms in atherosclerosis have been discussed in addition to intimal immune reactions which have been in focus. Even though the adventitial inflammation in atherosclerosis was noted already by Virchow and Rokitansky [83], and cellular immune infiltrates were described in the early 1960s [84], this phenomenon has only recently been more thoroughly studied [2,85]. The adventitia appears to be more complex than the intima and media also in normal arteries. Many cell types, including fibroblasts, dendritic cells, monocytes/macrophages, mast cells and T-cells are present. There are also nerve endings, small vessels (vasa vasorum), endothelial protenitor cells and the like in the intima, a matrix [86].

Data from mouse models of atherosclerosis are interesting, with B cells and T cells present in the adventitia forming inflammatory follicle-like structures [87]. An important role played by adventitial lymphocytes is suggested by several studies [2]. For example, proinflammatory IL-17A–producing T cells are present in the adventitia. Blockade of IL-17A led to a reduction in aortic macrophage accumulation and atherosclerosis [88].

Less is known about the role of the adventitia in human atherosclerosis,although immune competent cells including T cells and B cells are present in adventitial lymphoid follicles both in the aorta and in coronary arteries [2]. One important question is how the adventitia interacts with the intima in atherosclerotic lesions. It is interesting to note that B-cells are not common in the intima, as compared to findings from adventitia in atherosclerotic lesions. The role of B-cells in atherosclerosis most likely depends on subsets, at least according to studies in mouse models, with B2 lymphocytes being atherogenic and B1 lymphocytes protective against atherosclerosis [2].

Another factor in CVD, plaque rupture and late stage atherosclerosis may be intimal hemorrhage. Erythrocyte membranes are abundant in late stage lesions and could be a proinflammatory factor, increasing the risk of plaque rupture [89,90].

### Atherosclerosis and immunosenescence

An interesting question is whether atherosclerosis should be seen as a normal part of human aging or as a pathological process which could be, if not abolished, at least strongly reduced. Risk factors which cannot be modified include age and male sex, as opposed to those which can be favorably modified, such as hypertension, dyslipidemia, diabetes and smoking. One way to gain further insight into this intriguing question is to study atherosclerosis and CVD in populations living a life closer to the conditions during which humanity evolved. Further, studies of animals could provide important information.

The presence of atherosclerotic lesions in ancient Egyptian mummies was described more than 100 years ago and the findings were confirmed and extended in recent years; similar findings have been obtained in mummified bodies from other cultures as well [91]. It appears that these findings are clearer among wealthy individuals and, interestingly, were present at a relatively early age, since many of the mummies have been in their 40s and 50s when they died.

The famous ‘iceman’ (‘Ötzi’) found in South Tyrol in the early 1990s is of more interest as compared to the above-mentioned mummies. He died after being shot by an arrow, causing internal bleeding, 5,300 years ago in the Alps. Ötzi had atherosclerosis as determined by arterial calcifications [92]. His last meals consisted of meat (apparently from red deer and ibex) but also cereals [93]. Further, he seems to have been only approximately 45 years old when killed. Ötzi had signs of infection with Borrelia, which has been linked to atherosclerosis [73]. In a recent study his genome has been sequenced, and among other surprising findings is that he had a genetic predisposition for atherosclerosis [94].

A recent study of mummies from different geographic origins lends support to the notion that atherosclerosis was present among individuals in Stone Age cultures; for example, three of five hunter-gatherers had atherosclerosis [95]. It thus appears that atherosclerosis *per se* is not caused by a modern life-style, but is a part of human senescence. It is, therefore, interesting to study if the same applies to plaque rupture and CVD.

In Kitava, where New Guineans live a traditional life as horticulturalists, CVD and other conditions, such as Alzheimer’s disease and rheumatic disease, appear to be very rare. This is not likely to be explained simply by a low life expectancy allowing too little time for these conditions to become manifest [96,97]. The risk factor profile was favorable as compared to Westerners, with good metabolic control, lack of hypertension, somewhat but not strikingly better lipid profile and higher levels of anti-inflammatory natural anti-PC which appear to have anti-atherosclerotic properties. We also reported on infections studied in this population and suggested that infection with treponema is associated with the presence of atheroprotective anti-PC. It is possible that a low exposure in the West to such pathogens could contribute to low anti-PC levels, predisposing to western chronic inflammatory conditions, such as atherosclerosis [46]. Of note, treponema infections most likely have been with humanity since hominid times and, interestingly, anti-PC appears to play an important role in protection against this type of infection [98].

In our study on individuals from Kitava (New Guinea), the age span was between 40 and 86, with a mean age of 59.0, and here, traditional risk factors (except smoking!) were very favorable among Kitavans. This study lends support to the notion that CVD and, thus, plaque rupture is prevalent in modern societies. Further, immunosenescence may play an important role, since anti-PC decreased with age among Swedish controls, which was not the case with Kitavans [97].

A recent study of hunter-gatherers (pygmies living in the Cameroon forest) demonstrated lower aortic stiffness in this group [99], lending support to an atheroprotective role of the hunter-gatherer life style.

Still, it cannot be ruled out that low life expectancy among hunter-gatherers ‘masks’ CVD caused by atherosclerosis, although available evidence argues against this being an important factor, including an apparent lack of major risk factors, such as hypertension and diabetes. While mortality throughout life apparently is very high among hunter-gatherers, from causes such as violence and wars, accidents and infection, some also reached a high age [100].

Taken together, human studies indicate that atherosclerosis and its complications do not have to be a problem in normal aging up to high age, which would be a hopeful notion, implying that the disease at least could be postponed. Maybe such ‘natural’ atherosclerosis could be compared to cancer of the prostate, where many elderly men have signs of this disease, but will die from other causes.

It is interesting to note, that among wild animals, atherosclerosis has been described in elephants, which have a similar life span as humans and naturally large arteries. In one very interesting and unique study, almost 500 elephants were studied directly after death. The animals were randomly culled from free-living populations in East Africa as part of a program to decrease the elephant population to avoid starvation among these animals due to expansion of human agricultural activities. The elephant is interesting in this context, since its life span is similar to man. The study found atherosclerosis to be common, with necrosis, lipid accumulation, lymphocytic infiltration and calcification. Interestingly, there were no signs of mural thrombosis or plaque hemorrhage (even with lymphocytic infiltration) [101]. However, the degree to which CVD is a cause of death in elephants is not known.

### Anti-inflammatory and immune modulatory treatment against atherosclerosis and CVD

Even though the inflammatory nature of atherosclerosis and, thus, CVD has been known for a long time, there is no anti-inflammatory or immune modulatory treatment available to ameliorate these diseases. Further, to the best of my knowledge, there are no published convincing studies that such therapies work in humans. Here, I will discuss some immune-modulatory therapies that have potential against atherosclerosis.

#### Statins

Interestingly, statins, one of the most successful medicines in history from a commercial point of view, and clearly representing also a therapeutic improvement, may work to an unknown extent due to their pleiotropic, especially anti-inflammatory, properties. This effect is a ‘side-effect’ of these inhibitors of HMG CoA reductase, influencing prenylation as one mechanism. Further, the immunomodulatory effects of statins, directly interfering with major histocompatibility complex MHC class II presentation, have been described [102]. Interestingly, statins have been reported to be beneficial in RA; other anti-inflammatory mechanisms could be decreasing LDL-oxidation, and immune modulatory effects, decreasing MHC class II interaction with antigen [103]. In line with this, the Jupiter study demonstrated that statin treatment may be beneficial for individuals with raised high sensitivity CRP but normal LDL [104]. In a recent review by Ridker, it is stated that: ‘it is impossible in any statin trial to establish whether the clinical benefits of treatment are due to LDL-reduction alone, to inflammation inhibition, or to a combination of both processes’ [105]. It is striking that the possibility is raised that a novel and successful therapy developed to decrease cholesterol and specifically LDL, may work partly or even only, by another mechanism, namely inhibition of inflammation.

#### Anti-inflammatory treatment

Other interesting potential therapies include both unspecific and specific approaches. In RA, treatment with methotrexate weekly is routinely used, with beneficial effects on patients. A recent meta-analysis indicates that the risk of CVD is decreased in patients with RA treated with methotrexate [106]. Animal studies in which methotrexate decreases atherogenesis add support to methotrexate as a possible anti-inflammatory therapy in CVD [107]. In the Cardiovascular Inflammation Reduction Trial (CIRT) low dose methotrexate (target dose 20 mg/week) is tested for reduction of major CVD events among post-myocardial infarction patients with diabetes or metabolic syndrome [104].

Initially in RA, treatment with biologics, such as tumor necrosis factor (TNF)-inhibitors, has proven to be successful, and novel biologics also include inhibitors of other cytokines. However, there are different opinions as to whether biologics, such as anti-TNF, are beneficial from a cardiovascular point of view, although a recent study in which there was a decrease of CVD in RA adds support to this possibility [108,109].

The Canakinumab Anti-Inflammatory Thrombosis Outcomes Study (CANTOS) investigates if IL-1β inhibition could reduce the risk of MI, stroke, and cardiovascular death among stable coronary artery disease patients with high risk of CVD due to persistent elevations of CRP (≥2 mg/L) [105,110].

Other potentially interesting treatment moieties include inhibition of inflammatory lipid mediators, such as PAF [111]. We recently reported that Annexin A5, an anti-thrombotic plasma protein, is anti-inflammatory and inhibits atherosclerosis development and also improves endothelial function in a mouse model. Annexin A5 could thus represent another possible therapy [112]. Likewise, inhibition of PLs, such as lipoprotein associated PLA2, is of interest. Studies of the inhibition of Lp-PLA(2) activity with darapladib in patients after an acute coronary syndrome are under way [113].

#### Immune modulatory therapy

One example of possible immune modulatory, and thus potential anti-inflammatory, treatment against atherosclerosis and/or CVD is immune therapy against epitopes from oxLDL. In the mid-1990s, it was demonstrated that immunization with modified forms of LDL ameliorated atherosclerosis [114], which showed for the first time the possibility of inhibition of atherosclerosis development by immunization.

As discussed, during LDL-oxidation, various chemical moieties are formed, including both fragmented apoB and oxidized phospholipids. One approach is to target the apoB component. Peptides from apoB with promising immunomodulatory properties have been demonstrated to decrease atherosclerosis development in animal models [115-117]. However, a recent clinical study did not show any positive effect in humans [118]. The primary endpoint was the relative change in inflammatory activity in an index arterial vessel after twelve weeks, as measured by fluorodeoxyglucose-positron emission tomography/computed tomography (FDG-PET/CT) imaging. Further studies with other end points and using other, perhaps more established, techniques could clarify if this approach could still be promising in humans.

Another line of research and potential treatment is based on the phospholipid moiety as a target for treatment with monoclonal antibodies, specifically PC. As discussed above, this notion is supported by cohort studies, animal and *in vitro* experiments [37,39,40,42-44]. Mechanisms include anti-inflammatory [42,119], inhibition of cell death [44] and decreased uptake of oxLDL in macrophages [43].

A more unspecific method of immunomodulation is administration of Igs. This approach has shown promising results in animal studies with human Ig from pools of many donors, such as used in intravenous Ig treatment (IVIG) [120].

Yet another interesting possibility is to ameliorate atherosclerosis by immunomodulation with HSP. To the best of my knowledge, HSP-immunization demonstrated for the first time that atherosclerosis can be influenced by immunization. In a study using a rabbit model, such therapy increased atherosclerosis development [51].

Interestingly, nasal immunization with HSP65 led to induction of immune tolerance in a rabbit model, suggesting that immunization mucosally with Hsp65 protein could be a promising therapeutic method for atherosclerosis [121].

In another experiment, a vaccine was designed to target both HSP65 and cholesteryl ester transfer protein (CETP) in order to obtain both a positive effect on the immune reactions relevant in atherosclerosis and on blood lipids. Here, also, nasal immunization ameliorated atherosclerosis in a rabbit model [122].

There are also conflicting data. In a recent study, subcutaneous immunization with HSP-65 in apoE—mice actually reduced atherosclerosis [123]. Further, in another interesting study immunization with human HSP60 (with or without combination with apoB peptides) led to decreased atherosclerosis [124]. It is thus presently not clear how HSP-immunization could work as therapy against atherosclerosis development even in animal models. It is possible that differences in modes of presentation of the antigen could play a role. The potential anti-inflammatory treatments for atherosclerosis are summarized in Table 1.

**Table 1:** Potential treatment against inflammation in atherosclerosis

**Treatment**	**Targets**
Statins [102-105].	Prenylation, oxidation, MHC-class II presentation
Phospholipase-inhibitors [113] Annexin A5 [112]. Anti-PC [37,39,40,42-44].	Oxidized phospholipids
Anti-apoB [115-117].	apoB
Cytokine-inhibition [105,110]	Interleukin-1ß/Inflammasome
Metotrexate [105-107]	Inhibition of purine metabolism
Active immunization [40,114,115,121]	oxLDL, apoB, PC- epitopes, HSP

*anti-apoB*, antibody against apolipoprotein B; *anti-PC*, antibody against phosphorylcholine; *HSP*, heat shock protein; MHC, *oxLDL*, oxidized low density lipoprotein.

### Summary and conclusions

Taken together, atherosclerosis is the major underlying cause of CVD, which in turn is the major cause of death, at least in the developed world, and also an important cause of morbidity worldwide. During recent years, it has become clear that atherosclerosis is a chronic inflammation in large and middle-sized arteries, where activated immune competent cells are abundant. Inflammation could play a major role to trigger plaque rupture which is the immediate cause of CVD. Oxidized and/or modified forms of LDL, infections, HSPs and a more unspecific systemic inflammation are examples of hypotheses of underlying causes of the inflammation in atherosclerosis. Studies of anti-inflammatory and immune modulatory treatment are underway or discussed. Until such treatment studies in humans demonstrate that atherosclerosis is ameliorated, the causative role of inflammation in atherosclerosis remains a hypothesis.

**Figure 1:**
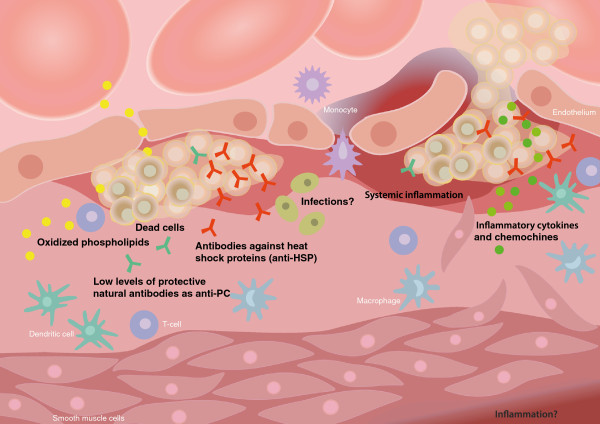
**Schematic illustration of an atherosclerotic plaque, with plaque rupture as a complication.** Potential underlying causes of inflammation and immune reactions are depicted. Illustration by Gunilla Elam.

The atherosclerotic lesion and potential causes of inflammation and immune reactions there are shown in Figure 1.

The normal artery has three parts or layers: 1) The tunica intima which is lined with endothelial cells in contact with the blood stream and also, in humans, containing some SMC. 2) The media which contains SMC and extracellular matrix. 3) The adventitia which appears to be more complex than the other two layers, although the exact role of the adventitia in atherosclerosis is not known. Many cell types, including fibroblasts, dendritic cells, monocytes/macrophages, mast cells and T-cells are present. There are also nerve endings, small vessels (vasa vasorum), endothelial protenitor cells, and a matrix.

During atherosclerosis development early steps include activation of the monolayer of the endothelial cells with expression of adhesion molecules and migration of blood monocytes/macrophages, dendritic cells, T cells and some B-cells into the intima, and also adhesion of modified forms of LDL to matrix components. Monocytes/macrophages are especially numerous and develop into foam cells, filled mainly with modified LDL. Medial SMC migrate into the intima and develop a more synthetic phenotype, producing matrix components. As the atherosclerotic plaque grows, a fibrous cap is developed to cover it. Later on, dying and dead cells accumulate, many of which are derived from foam cells. With further advancement, microvessels develop in the plaque. A feared complication of atherosclerosis, which by itself can be seen as a part of normal aging, is fracture and/or rupture of the fibrous cap, leading to contact between the blood coagulation components and plaque material including tissue factor, which triggers thrombosis, leading to infarction.

The cause of the inflammation and ensuing plaque rupture has not been clarified, although non-mutually exclusive possibilities exist. OxLDL as opposed to LDL, activate T-cells, and stimulate monocyte/macrophages and other cell types of the plaque. Part of this effect is likely mediated by inflammatory phospholipids. Natural anti-PC and other phospholipids could be protective by neutralizing the inflammatory effects, and low levels of these could be a cause of inflammation.

HSPs induced in the plaque by stress, oxLDL and other factors could become immunogenic, and trigger immune responses which could both induce and potentiate inflammation in atherosclerosis. Infections have been much discussed, and many infectious agents are present in lesions. Even though they potentially could be a cause of atherosclerosis, this is not supported fully by available evidence, and they could also be innocent bystanders. As a proximate cause of plaque rupture, cytokines and chemokines could play a major direct role, induced by other factors.

## Abbreviations

AA: Aggregatibacter actinomycetemcomitans; AGEs: Advanced glycation end products; anti-apo B: Antibodies against apoprotein B; anti-PC: Antibodies against phosphorylcholine; aOxCL: Antibodies against oxidized forms of cardiolipin; aOxPS: Antibodies against oxidized phosphatidylserine; beta2GPI: Beta 2-glycoprotein I; CMV: Cytomegalovirus; CP: *Chlamydia pneumoniae*; CRP: C-reactive protein; CVD: Cardiovascular disease; DAMP: Danger associated molecular patterns; EBV: Epstein-Barr virus; HP: *Helicobacter pylori*; HSP: Heat-shock proteins; Ig: Immunoglobulin; IL: Interleukin; IVIG: Intravenous immunoglobulin; LDL: Low density lipoprotein; LPC: Lysophosphatidylcholine; MDA: Malondialdehyde; MI: Myocardial infarction; oxLDL: Oxidized low density lipoprotein; PAF: Platelet-activating factor; PAMP: Pathogen associated molecular patterns; PC: Phosphorylcholine; PG: *Porphyromonas gingivalis*; PLA2: Phospholipase 2; RA: Rheumatoid arthritis; SMC: Smooth muscle cells; SLE: Systemic lupus erythematosus; TNF: Tumor necrosis factor.

## Competing interests

JF is named as inventor on patent applications relating to phospholipids and antibodies.

## Pre-publication history

The pre-publication history for this paper can be accessed here:

http://www.biomedcentral.com/1741-7015/11/117/prepub
